# Bioavailable Menthol (Transient Receptor Potential Melastatin-8 Agonist) Induces Energy Expending Phenotype in Differentiating Adipocytes

**DOI:** 10.3390/cells8050383

**Published:** 2019-04-26

**Authors:** Pragyanshu Khare, Aakriti Chauhan, Vibhu Kumar, Jasleen Kaur, Neha Mahajan, Vijay Kumar, Adam Gesing, Kanwaljit Chopra, Kanthi Kiran Kondepudi, Mahendra Bishnoi

**Affiliations:** 1 National Agri-Food Biotechnology Institute (NABI), Knowledge City-Sector 81, SAS Nagar, Punjab 140603, India; khare.pragyanshu@gmail.com (P.K.); chauhanaakriti11@gmail.com (A.C.); vibhu.kumar99@gmail.com (V.K.); jasleen.820@gmail.com (J.K.); mahajan23neha@gmail.com (N.M.); 2210.vijay@gmail.com (V.K.); kiran@nabi.res.in (K.K.K.); 2 Pharmacology Division, University Institute of Pharmaceutical Sciences (UIPS), Panjab University, Chandigarh 160014, India; dr_chopra_k@yahoo.com; 3 Department of Pharmacology & Toxicology, National Institute of Pharmaceutical Education and Research (NIPER)-Raebareli, Transit campus Lucknow, Uttar Pradesh 226301, India; 4 Regional Centre for Biotechnology, Faridabad-Gurgaon expressway, Faridabad, Haryana 121001, India; 5 Department of Biotechnology, Panjab University, Sector-25, Chandigarh 160014, India; 6 Department of Endocrinology of Ageing, Medical University of Lodz, Zeligowski St, No 7/9, 90-752 Lodz, Poland; adges7@yahoo.com

**Keywords:** adipose tissue, bioavailable, menthol, topical, TRPM8

## Abstract

Recent evidence supports the role of menthol, a TRPM8 agonist, in enhanced energy expenditure, thermogenesis and BAT-like activity in classical WAT depots in a TRPM8 dependent and independent manner. The present study was designed to analyse whether oral and topical administration of menthol is bioavailable at subcutaneous adipose tissue and is sufficient to directlyinduce desired energy expenditure effects. GC-FID was performed to study menthol bioavailability in serum and subcutaneous white adipose tissue following oral and topical administration. Further, 3T3L1 adipocytes were treated with bioavailable menthol doses and different parameters (lipid accumulation, “*browning*/*brite*” and energy expenditure gene expression, metal analysis, mitochondrial complex’s gene expression) were studied. No difference was observed in serum levels but significant difference was seen in the menthol concentration on subcutaneous adipose tissues after oral and topical application. Menthol administration at bioavailable doses significantly increased “*browning/brite*” and energy expenditure phenotype, enhanced mitochondrial activity related gene expression, increased metal concentration during adipogenesis but did not alter the lipid accumulation as well as acute experiments were performed with lower dose of menthol on mature adipocytes In conclusion, the present study provides evidence that bioavailable menthol after single oral and topical administration is sufficient to induce “*brite*” phenotype in subcutaneous adipose tissue However, critical dose characterization for its clinical utility is required.

## 1. Introduction

The imbalance between energy intake and energy expenditure is a well-known aspect of the escalating prevalence of obesity throughout the world as a major nutritional challenge [1,2]. Multiple factors like physical activity, dietary energy intake, basal metabolic rate, variation in environmental and body temperature are reported to influence energy expenditure [3,4]. Enhancement of energy expenditure is one of the cardinal approach toward prevention of body weight gain and obesity [5]. Numerous studies have reported the direct relation of cold exposure with non-shivering thermogenesis, followed by reduction in body fat [6,7,8]. Interestingly, clinical studies have also reported a vital link between cold-exposure and elevated energy expenditure through adaptive thermogenesis [9]. Studies on adipocytes have also reported a relationship between cold receptor, TRPM8 and energy expenditure showing upregulated expression of UCP-1 and PGC-1α owing to TRPM8 activation [10].

Complex physiology of the body makes it difficult to understand the exact mechanisms involved in energy expenditure. Transient receptor potential cation channel subfamily Melastatin member 8 (TRPM8) is a thermoreceptor present on the sensory nerve endings innervating the gut and skin [11,12,13] that senses non-noxious cold stimuli ranging from 18–25 °C, hence making it attractive target for cold mimicking. Involvement of TRPM8 dependent and independent pathways in mediating cold-induced energy expenditure is well reported. Recently we have published that anti-obesity effect of menthol, a TRPM8 agonist is mediated through glucagon dependent mechanisms particularly in adipose tissues [14]. Activation of TRPM8 receptors in the gut and skin by oral and topical administration of menthol leads to increase in serum glucagon levels, thus activating several downstream catabolic processes like glycogenolysis, gluconeogenesis, *browning* of white adipose tissue (WAT) and activation of energy expenditure markers in WAT and brown adipose tissue (BAT) [14]. This is supposedly an indirect action of menthol on adipose tissue wherein presence of TRPM8 is not essential. On the other hand, studies from our laboratory provide a strong evidence of significant expression of TRPM8 in undifferentiated (pre-adipocytes) and differentiated (adipocytes) 3T3-L1 cells suggesting its significance in adipogenesis [15]. Also, TRPM8 is functionally expressed in rodent WAT [16] and BAT [17] as well as human WAT [18]. Alternatively, Ma et al. showed that the mouse BAT expresses TRPM8 receptor which upon activation by menthol, significantly increased the levels of p-PKA that further phosphorylates the transcription factor cyclic AMP-responsive element-binding protein (CREB), ultimately leading to enhanced UCP1 expression [17,19]. Thus, chronic dietary menthol increased thermogenesis, core body temperature, prevented diet-induced obesity and the abnormal glucose homeostasis in wild-type mice but not in UCP1^(−/−)^ and TRPM8^(−/−)^ mice [17], suggesting TRPM8 receptor presence on adipose tissue is essential. Other studies also demonstrated the induction of white-to-brown-like phenotype through TRPM8 activation on human WAT [18,20], summarizing the importance of the presence of TRPM8 on adipose tissue. 

We hypothesize that different TRPM8 dependent and independent actions are contributing to the anti-obesity effect of menthol. The present study is an attempt to reveal whether topical (10% *w*/*v*) or oral (200 mg/kg, p.o.) administration of menthol used during acute studies in our previous work is bioavailable at subcutaneous adipose tissue (direct or through serum). Further, using in-vitro 3T3L1 cell line model, we attempted to study that whether bioavailable menthol at subcutaneous adipose tissue is sufficient to induce direct effect on “*browning*” and mitochondrial energy metabolism followed by enhanced energy expenditure effects. Furthermore, this manuscript endeavours to elucidate the presence of metal ions and their role in mitochondrial electron transport chain for significant elevation in energy expenditure owing to the menthol treatment. 

## 2. Materials and Methods

### 2.1. Chemicals

Mouse 3T3-L1 preadipocytes were obtained from National Centre for Cell Science (NCCS, Pune, India). Dulbecco’s Modified Eagle Medium (DMEM), Foetal Bovine Serum (FBS), Penicillin-Streptomycin and Phosphate-Buffered Saline (PBS) was procured from Lonza Inc. (Walkersville, MD, USA). Menthol, AMTB hydrate, Isopropyl alcohol (IPA), 3-isobutyl-1-methylxanthine (IBMX), Dexamethasone (DMS), Insulin, Oil-Red-O (ORO) powder, 3-(4,5-dimethylthiazol-2-yl)-2,5-diphenyl-2*H*-tetrazolium bromide (MTT)and Dimethyl Sulfoxide (DMSO) were obtained from Sigma Aldrich Co. (St. Louis, MO, USA). GeneZol RNA Extraction Reagent was obtained from Genetix Biotech Asia Pvt. Ltd. (New Delhi, India). Absolute Ethanol (Biotechnology grade) was procured from Amresco Inc. (Cochran Road, Solon, OH, USA). Nitric acid (Trace Metal Grade) was obtained from Fisher Scientific UK Limited (Bishop Meadow Road, Loughborough, Leicestershire, UK) and Dichloromethane (DCM) was procured from Central Drug House (New Delhi, India). All other reagents used were of analytical grade and were procured from local supplier.

### 2.2. Animal Treatment

21 Swiss Male Albino mice (25–30 g) bred in Central Animal Facility of National Institute of Pharmaceutical Education and Research (NIPER), Mohali, Punjab, India were used for the experiment. 3 mice per cage were housed on a 12 h light/dark cycle with ad libitum food and water supply. The experiment was approved by the Institutional Animal Ethical Committee, NIPER, Mohali, Punjab, India. All the experiments were conducted in accordance with the Committee for the Purpose of Control and Supervision in Experiments on Animals (CPCSEA) guidelines on the use and care of experimental animals. Mice were randomly divided into 3 different groups consisting 3 animals for control group, 9 animals for oral administration of menthol and 9 animals for topical administration of menthol. Oral administration of menthol was given at the dose of 200 mg/Kg in 0.1% tween-80, while topical administration of menthol was given on abdominal area by 1 mL topical application of 10% menthol in 0.1% tween 80. Animals were euthanized after 30, 60 and 120 min of menthol administration by cervical dislocation, blood was collected by cardiac puncture for serum isolation and subcutaneous white adipose tissues were collected for pharmacokinetic profiling of menthol [14].

### 2.3. Gas Chromatography-Flame Ionization Detector (GC-FID) for Menthol Bioavailability in Serum and Subcutaneous WAT

#### 2.3.1. Extraction Procedure

Both serum and adipose tissue samples were extracted fromDCM containing 200 ng/mL of thymol as internal standard. The samples were continuously vortexed for 3 min and centrifuged at 6000 rpm for next 3 min. After centrifugation, the supernatant was isolated and again extracted in DCM as discussed above. The supernatant finally obtained was used for the pharmacokinetic evaluation of menthol both in serum and adipose tissue.

#### 2.3.2. GC-FID Protocol

Separation and detection was done by injecting 1 µL of target analyte in gas chromatograph (Agilent 7890B) equipped with split less injector system and flame ionization detector. Ultra-pure nitrogen gas was passed through a molecular sieve and oxygen trap and was used as carrier gas with flow rate of 1 mL/min. The injection port was held at 240 °C operated at split less mode. Separation was done using DB5 capillary column (Agilent Technologies) with dimension 30 m × 320 µm × 1 µm with oven temperature kept at 40 °C and then increased to 250 °C at the rate of 10 °C/min, hold time for 2 min. and sample run time for 23 min. the FID temperature was maintained at 280 °C for detection. Flow of hydrogen gas used for FID was at the rate of 30 mL/min. The flow of air for FID was 300 mL/min along with makeup flow at the rate of 25 mL/min. Calculations were done using EZ Chrome Software (Menlo Park, CA, USA).

### 2.4. 3T3-L1 Cell Culture

Mouse 3T3-L1 pre-adipocytes were allowed to culture in basal medium comprising DMEM supplemented with 10% *v*/*v* FBS and 1% penicillin-streptomycin. Whole cell culture was maintained at 37 °C in a humidified incubator along with continuous supply of 95% O_2_ and 5% CO_2_. At 60% confluency, cellular differentiation from 3T3-L1 pre-adipocytes to adipocytes was induced using differentiation medium containing basal medium supplemented with 0.1 mM IBMX, 0.1 µM DMS and 1 µg/mL insulin for 48 h. Stock solutions of 0.1 µM DMS and 0.1 mM IBMX were prepared in absolute ethanol and DMSO respectively and were directly supplemented to DMEM culture medium. Further, the cells were shifted to maintenance medium (basal medium with 1 µg/mL insulin) for 10 days with medium replacement on every alternate day. Working solutions of 1, 50, 100, 200 and 300 µM menthol for the treatment were also prepared in absolute ethanol and directly supplemented to culture (differentiation/maintenance) medium during adipogenesis. Dose selection of menthol for in-vitro study was done on the basis of in-vivo pharmacokinetic profile of menthol in serum and adipose tissues. Two different sets of experiments were performed, (A) Effect of menthol administration at different doses (1, 10, 30 and 50 µM during adipogenesis. (B) Effect of menthol administration (1 µM) on matured adipocytes after 1 h of treatment. The entire cell culturing experiments were carried out as per American Type Culture Collection (ATCC) guidelines.

### 2.5. Cell Viability Assay 

Cell cytotoxicity was assessed using MTT assay. Undifferentiated 3T3-L1 preadipocyte cells were seeded in a 96 well plate at density of 1 × 10^4^ cells/well and incubated with varying concentrations (50, 100 and 300 µM) of menthol dissolved in <0.001% ethanol at 37 °C for 24 h. For control, undifferentiated pre-adipocytes treated with ethanol (<0.001%) were taken. After 24 h, media was replaced with MTT as per the instructions by manufacturer (Sigma Aldrich Co., St. Louis, MO, USA). Formazan crystals formed by live cells are solubilized in isopropanol and absorbance was measured at 570 nm using microplate reader (Spectra max M5e, Molecular Devices LLC, San Jose, CA, USA) [21].

### 2.6. Oil-Red-O Staining

A stock solution of 0.5% *w*/*v* of ORO was prepared IPA and was stored at room temperature. A working solution of ORO stain was prepared by diluting the stock solution with distilled water in the ratio 3:2. For staining, the supernatant in the culture plates after treatment with varying concentrations of menthol (1, 50 and 200 µM) was removed and cells were washed using PBS. After washing, mature adipocytes were fixed with 10% *v*/*v* formaldehyde in PBS and kept for 30 min at room temperature followed by three subsequent washing with PBS. Then, the PBS was aspirated and 60% of IPA was added followed by incubation for 5 min. Further, the cells were stained in ORO working solution filled in culture plates to completely cover the plate bottom (1.25 mL per well for 6-well culture plate) and kept for incubation for 10 min. The cells were then washed with 60% IPA to rinse away excess ORO dye followed by final elution of dye in 100% IPA (2.5 mL per well for 6-well culture plate). The plates were again kept for incubation at room temperature for 10 min. Now, 200 µL of each eluate was added to a 96 well microtiter plate. Also, 200 µL of 100 % IPA was filled on the same microtiter plate as blank. Finally, intracellular lipid content was quantified after extracting ORO bound cells with 100% IPA and absorbance was taken at 570nm using Nano Quant–Infinite M200 Pro (Tecan, Switzerland) [22,23]. 

### 2.7. ICP-MS for Metal Analysis

Cells from control and test samples (1, 50 and 200 µM administration of menthol during differentiation) were pellet down and digested with nitric acid using Microwave Reaction System (Mars 6, CEM Corporation, Matthews, NC, USA). The concentration of different elements was estimated in the digested samples using standard protocol for inductively coupled plasma mass spectrometry (ICP-MS:77006, Agilent Technologies, Santa Clara, CA, USA) [14]. 

### 2.8. qRT-PCR for Browning/Energy Expenditure Genes

Total RNA from both control and treated adipocytes was extracted after 10 days of treatment using Acid guanidinium thiocyanate-phenol-chloroform extraction method with GeneZol RNA Extraction Reagent, IPA, Chloroform and was reversely transcribed to cDNA from 1 µg of RNA using iScript cDNA Synthesis Kit (Bio-Rad Laboratories Inc., Hercules, CA, USA). Quantitative real time-polymerase chain reaction (qRT-PCR) was performed with the cDNA as templates in 40 cycles using Custom RT^2^ PCR Array Kit (CLAM 30615C, Qiagen Lifesciences, Waltham, MA, USA) and RT^2^ SYBR Green ROX qPCR Mastermix (Qiagen Lifesciences) keeping GAPDH as internal control and 7 gene specific primers for adipogenesis including *TNFRSF9, PPARGC1A, PRDM16, CIDEA, TLE3, HOXC10* and *NRIP1*. The qRT-PCR process was performed by programming thermocycler (CFX96 Touch Real-Time PCR Detection System (Bio-Rad Laboratories Inc., Hercules, CA, USA)) to apply initial cycle for denaturation at 95 °C for 2 min, followed by 40 cycles of annealing and elongation at 60 °C for 30 s and denaturation at 95 °C for 5 s. The complete experiment was performed using three biological replicates [14]. Analysis of relative gene expression was done using 2^−ΔΔct^ method [24] and values were expressed in the terms of fold change with reference to control [14].

### 2.9. Gene Expression Analysis of Mitochondrial Energy Metabolism

Total RNA from both control and treated adipocytes was extracted after 10 days of treatment using Acid guanidinium thiocyanate-phenol-chloroform extraction method with GeneZol RNA Extraction Reagent, IPA, Chloroform and was reversely transcribed to cDNA from 5 µg of RNA using RT^2^ first strand kit (Qiagen Lifesciences). qRT-PCR was performed using RT^2^ Profiler PCR Array Kit for Mouse Mitochondrial Energy Metabolism (PAMM 008ZC-12, Qiagen Lifesciences, Waltham, MA, USA) and RT^2^ SYBR Green ROX qPCR Mastermix (Qiagen Lifesciences). The thermocycler (Applied Biosystem 7500F Real Time PCR System (Applied Biosystems, Fosters City, CA, USA)) was programmed to apply initial cycle for denaturation at 95 °C for 10 min, followed by 40 cycles of annealing and elongation at 60 °C for 1 min and denaturation at 95 °C for 15 s. Three biological replicates were used for this experiment [14]. Analysis of relative gene expression was done using 2^−ΔΔct^ method [24] and values were expressed in the terms of fold change with reference to control [14].

### 2.10. Statistical Analysis

All the values are expressed as mean ± SEM. A group comparison in in-vivo study was carried outusing unpaired student t-test and two-way ANOVA followed by Tukey’s post-hoc test. Group comparison in-vitro study was done using unpaired student t-test and one-way ANOVA followed by Tukey’s post-hoc test. Pearson correlation was performed and representative matrix was made using Gitools 2.3.1. (San Fernando Rd, Burbank, CA, USA). *p* ≤ 0.05 was considered statistically significant. Correlation matrix and clustering was done by k-means ++ Manhattan distance clusters 6.

## 3. Results

### 3.1. Pharmacokinetic Profile of Acute Oral Administration and Topical Application of TRPM8 Agonist (Menthol) in Mice

**Figure 1:**
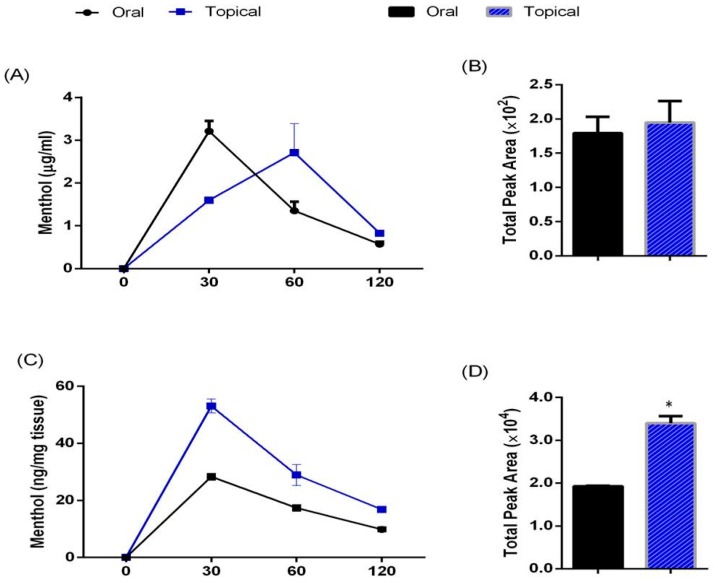
Pharmacokinetic of menthol administration upon oral and topical administration(**A**) Time dependent changes in serum concentration of menthol; (**B**) Area under the time-concentration curve in serum of menthol treated mouse; (**C**) Time dependent changes in concentration of menthol in subcutaneous adipose tissue; (**D**) Area under the time-concentration curve in subcutaneous adipose tissue of menthol treated mice. Oral- Single dose of oral menthol administration at 200 mg/kg. Topical-Single dose of topical menthol application at 10% concentration (300 µL/animal). *n* = 3. All the values are expressed as mean ± S.E.M; * *p* < 0.05.

Serum menthol concentration after oral (200 mg/kg) and topical administration (10% *w*/*v*) was determined at different time intervals of 0, 30, 60 and 120 min. The peak plasma concentration after oral and topical administration was attained at 30 and 60 min respectively. Bioavailable concentration of menthol in serum after oral administration at 30, 60 and 120 min was found to be 3.28 µg/mL (20.4 µM), 1.51 µg/mL (9.66 µM) and 0.76 µg/mL (4.86 µM) respectively. Serum concentration after topical administration at 30, 60 and 120 min was found to be 1.60 µg/mL (10.23 µM), 2.71 µg/mL (17.28 µM) and 0.82 µg/mL (5.2 µM) respectively (Figure 1A). Area under the curve of concentration and time graph was also determined and no significant difference was observed in serum metal concentration between oral and topical administration (Figure 1B).

Similarly, menthol concentration in the adipose tissue was also determined at the same time intervals. The peak concentration after oral and topical administration was attained at 30 min. Concentration of menthol after oral administration at 30, 60 and 120 min was found to be 28.33 µg/mL (181.09 µM), 17.38 µg/mL (111.2 µM) and 12.51 µg/mL (79.98 µM) respectively. Menthol concentration in the adipose tissue after topical administration at 30, 60 and 120 min was found to be 53.09 µg/mL (339.74 µM), 28.94 µg/mL (185.19 µM) and 20.11 µg/mL (128.69 µM) respectively (Figure 1C). Area under the curve of concentration and time graph for topical administration was significantly higher than the oral administration which signifies that higher amount of menthol was available in the adipose tissue after topical administration (Figure 1D).

### 3.2. Menthol Treatment Did Not Affect Cellular Differentiation, Lipid Accumulation and Cell Viability in 3T3-L1 Cells In-Vitro

**Figure 2:**
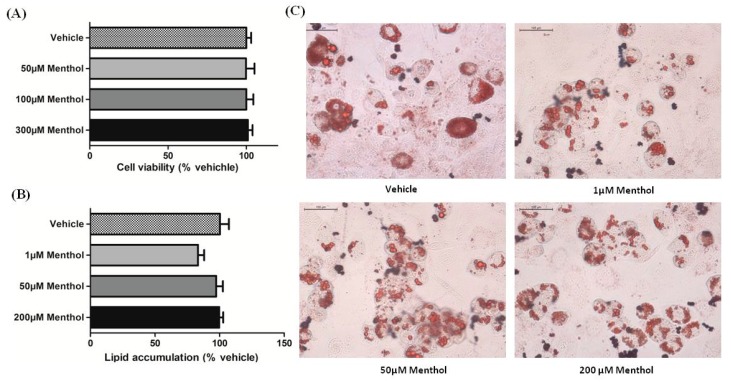
Effect of menthol on adipogenesis in 3T3-L1 cells. (**A**) Cell viability in pre-adipocytes treated with menthol for 24 h. (**B**) Effect of menthol on lipid accumulation in 3T3-L1 cells. (**C**) Effect of various concentration of menthol on differentiation of 3T3-L1 cells. Red spots in images represents area stained by Oil Red O dye. All values are expressed as mean ± S.E.M. (*n* = 3). One-way ANOVA followed by Tukey’s multiple comparison post hoc test was applied. * each vs. vehicle.

No change in cell viability was observed with menthol treatment in comparison to the vehicle treated group up to 300 µM dose (Figure 2A). Oil red O staining is used as a quantitative method to analyse the difference in the degree of differentiation and lipid accumulation in adipocytes in cell culture. The effect of varying concentrations of menthol (1 µM, 50 µM, 100 µM and 200 µM) was evaluated during adipogenesis. Slight decrease in lipid accumulation was observed with menthol 1 µM dose as compared to vehicle treated group and no change was observed with 50 and 200 µM doses (Figure 2B). No significant difference in cellular differentiation of 3T3-L1 cells was observed between the vehicle and menthol treated cells (Figure 2C).

### 3.3. Menthol Treatment Altered Metal Concentration and Browning Gene Markers in 3T3-L1 Pre-Adipocytes In-Vitro 

**Figure 3:**
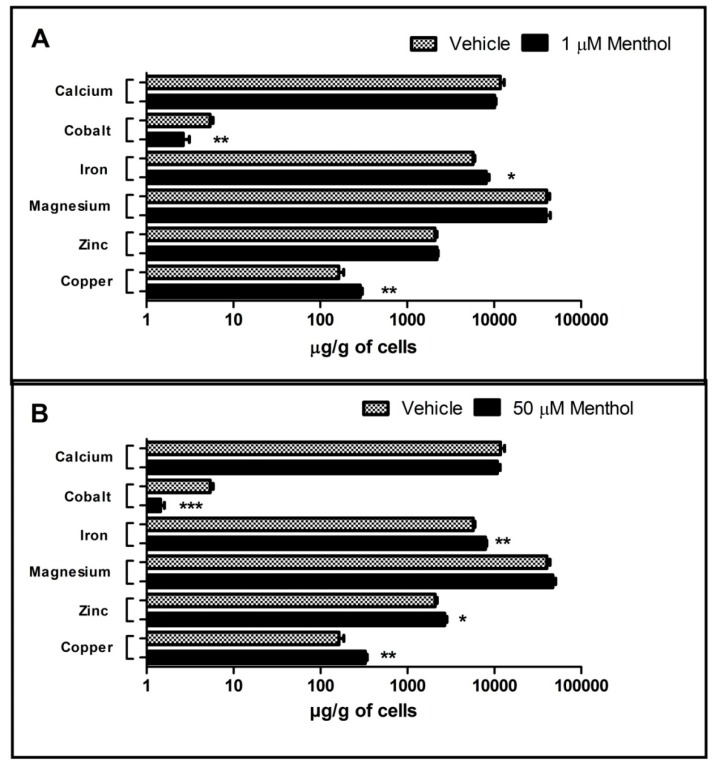
Effect of menthol treatment on. (**A**) Metal concentration (Ca, Co, Fe, Mg, Zn, Cu) in 3T3-L1 cells at 1 μM dose; (**B**) Metal concentration (Ca, Co, Fe, Mg, Zn, Cu) in 3T3-L1 cells at 50 μM dose; *n* = 3–5; All the values are expressed as mean ± S.E.M; Analysis was carried out by student unpaired *t*-test; * *p* < 0.05, ** *p* < 0.01, *** *p* < 0.001.

The effect of 1, 50 and 200 (data not shown) µM concentrations of menthol treatment during the adipogenesis process on different concentrations of metals in matured adipocytes was evaluated by ICP-MS. Significant increase in the concentrations of iron and copper was observed after 1 µM menthol treatment as compared to their respective controls. Similar pattern was also observed with 50 µM menthol treatment, showing marked elevation in levels of iron, copper and zinc as compared to their corresponding controls. However, significant reduction in cobalt metal levels was observed with both doses of menthol (1 and 50 µM) as compared to vehicle treated group. No significant change in the level of metals like calcium and magnesium was observed with both doses of menthol (Figure 3A,B). There was no significant difference in metal concentrations at 50 µM and 200 µM doses of menthol, hence we used 1 and 50 µM for further studies. 

**Figure 4:**
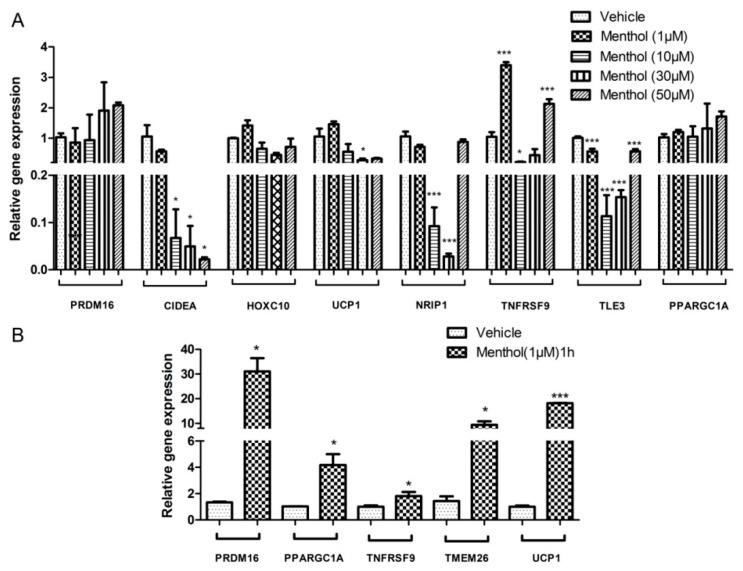
Effect of menthol treatment on: (**A**) Relative expression of energy expenditure genes in 3T3-L1 cells at 1 μM, 10 μM, 30 µM and 50 μM menthol treatment; (**B**)Relative expression of energy expenditure genes in 3T3-L1 cells at 1 μM menthol treatment for 1 h. *n* = 3–5; All the values are expressed as mean ± S.E.M; Analysis was carried out by student paired *t*-test and one-way ANOVA followed by Tukey’s multiple comparison; * *p* < 0.05, ** *p* < 0.01, *** *p* < 0.001.

Treatment with 1, 10, 30 and 50 µM concentrations of menthol was given during the process of adipogenesis to check the mRNA expression of *browning* markers. At 1 µM menthol increase in the mRNA expression of *browning* markers that is, Peroxisome proliferator-activated receptor gamma (*PPARCG1A*), Homeobox C10 (*HOXC10*), Tumour necrosis factor receptor superfamily member 9 (*TNFRSF9*) and Uncoupling protein (*UCP1*) was observedas compared to vehicle treated group. A marked reduction in the mRNA expression of Transducin like enhancer of split 3 (*TLE3*) and no change was observed in PR domain containing16 (*PRDM16*), Cell Death inducing DFFA like effector A (CIDEA) and Nuclear receptor-interacting protein 1 (*NRIP1*) at 1 µM menthol treatment. At 10 µM, there was a significant reduction in gene expression of *CIDEA*, *NRIP1*, *TLE3* and *TNFRSF9*. No significant change in levels of *PRDM16, HOXC10*, *UCP1* and *PPARGC1A*, similar pattern of change in levels of gene expression was observed at 30 µM. Although at 50 µM menthol treatment significantly reduced the mRNA expression of *CIDEA* only. There was no significant difference observed in expression of *HOXC10*, *PRDM16*, *UCP1*, *NRIP1* and *PPARGC1A* as compared to control (Figure 4A). Acute experiment was performed at 1 µM concentration of menthol for 1 h on mature adipocytes. Significant increase in the gene expression of *browning* markers that is, *PRDM16*, *PPARGC1A*, *TNFRSF9*, *TMEM26* and *UCP1* was observed (Figure 4B).

### 3.4. Menthol Treatment Modulated Mitochondrial Activity Gene Markers for Energy Metabolism in 3T3-L1 Pre-Adipocytes In-Vitro

**Figure 5:**
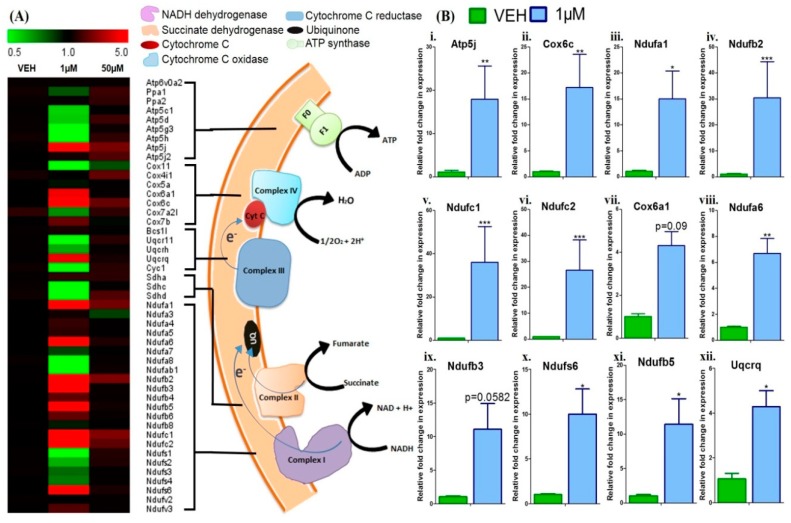
Heat map analysis of gene expression; and genes with statistically significant differential expression in 1 µM menthol treated group. (**A**) Heat map shows gene expression in vehicle, 1 µM and 50 µM menthol treated 3T3-L1 cells (*n* = 3 each). Colour from red to green indicates high to low expression. (**B**) Comparative analysis of selected genes in 1 µM menthol treated group. Normalization was done with reference gene B2M (β-2-microglobulin). The relative fold change in gene expression for mitochondrial biogenesis genes was compared in the three groups. Statistical analysis was done for genes i-vi using two-way ANOVA and for genes vii-xii using two-tailed unpaired t-test. * *p* <0.05, ** *p* <0.01, *** *p* <0.001, each vs vehicle treated group.

The effect of 1 µM and 50 µM of menthol treatment on different mitochondrial genes was studied. In the NADH dehydrogenase (Complex I), significant increase in the gene expression of *Ndufa1*, *Ndufb2, Ndufc1, Ndufc2, Ndufa6, Ndufb3, Ndufb5, Ndufs6* was observed as compared to vehicle treated group. Significant increase in expression of *Uqcrq* gene in cytochrome c reductase (complex III) was observed as compared to vehicle treated group. In the cytochrome c oxidase (complex IV) significant increase in expression of *Cox6a1, Cox6c* genes were also observed. In the F-Type ATP synthase (complex V) significant elevation in the *Atp5j* gene expression was also observed as compared to vehicle treated group (Figure 5A,B). Although, 50 µM menthol supplementation also displayed a modulatory effect over gene expressions for mitochondrial energy metabolism, however, the change observed was insignificant in comparison to vehicle treated group (Figure 5A).

## 4. Discussion

In our recently published manuscript, we have shown that the oral and topical administration of menthol, a TRPM8 agonist, has anti-obesity potential through a TRPM8 mediated glucagon dependent mechanism [14]. We provided evidence that TRPM8 mediated increase in serum glucagon and resultant increase in “glucagon machinery” in liver and adipose tissue is the signature of global shift from “fat storing state” to “fat burning state” in response to menthol administration [14]. During this work, we could suggest that the presence of TRPM8 receptor on adipose tissue is not required and the effect is selective to the glucagon receptor present on adipose tissue, hence we may say that it is an indirect action of menthol on adipose tissue. Also, recent paper by Clemmemsen and colleagues suggested that icilin, a TRPM8 agonist, effect on BAT energy expenditure cannot be explained by direct effects of icilin on adipocytes suggesting indirect actions to increase thermogenesis, likely through induction of sympathetic tone [25]. However, the literature provide evidence for the presence of functional TRPM8 receptors on mouse and human adipose tissue, both white and brown [16,17,18,26] and adipocyte cell lines [15]. These studies have mentioned that menthol induced increase in calcium influx in adipose tissue, mitochondrial activation and enhanced gene expression is mediated by TRPM8 receptors present on adipose tissue. The doses used in these studies are not based on the bioavailability profile of menthol, hence to establish a link between bioavailable doses of menthol and its functional effects on adipocytes is warranted. In this work, we tend to answer the question that at the acute doses showing anti-obesity effect [14], how much of menthol was bioavailable in serum and subcutaneous WAT. Also, whether this bioavailable menthol has any direct action on adipose tissue mediated through TRPM8 or others to induce energy expenditure. 

Menthol was bioavailable to adipose tissues via both routes, oral and topical, through serum and direct absorption, respectively. Serum concentration of menthol reached a maximum at 30 min and 60 min respectively in the case of oral and topical administration; however, the total area reached under the curve/peak concentration was similar over a period of 2 h. The concentration of menthol in subcutaneous WAT reached a maximum at 30 min in the case of both oral and topical administration, with a maximum peak concentration significantly higher in the case of topical administration. We can easily argue that due to proximity of subcutaneous WAT to the site of application (topical), we see a significantly higher concentration. Menthol is lipophilic in nature and its partitioning in adipose tissue through topical application is significantly higher as compared to oral administration. We understand that partitioning in rodent adipose tissue is positively predictive of partitioning in human adipose tissue [27], hence we suggest that this data has clinical utility through the direct effect of menthol on adipose tissue [14]. Previous literature also supports that L-menthol administration increases metabolic rate and thermogenesis in humans [27]. In the same study, authors have concluded that these effects are minor in oral administration as compared to topical administration due to faster metabolism of menthol (glucoronidation) and higher values of menthol glucuronides levels in blood [27]. Summarizing, route of administration is important for the action of menthol on subcutaneous adipose tissue whereas in serum both oral and topical are similarly bioavailable. 

Now, the question is whether bioavailable menthol after topical administration is enough to show the desired effects in adipose tissue, may be directly. We used pharmacokinetic based mathematical calculations to convert the bioavailable concentration of menthol in µM concentrations. We calculated that there is a range of concentration that is, 1 µM to 200 µM which will sufficiently cover the bioavailable amount of menthol on adipose tissues after topical administration. At these concentrations of menthol, 3T3L1 cells were viable as assessed by MTT assay. Also, at these doses there was no significant change in the accumulation of fat in differentiating adipose cells as shown by ORO staining. There was minor observation that some of cells were of smaller sizes. 

There is lot of recent literature linking adipose tissue metal concentration with adipose tissue health, differentiation and “*browning*” of white adipose tissue. Specifically, in this regard, iron and copper has major significance [28,29]. Iron and copper are essential components of the mitochondrial inner membrane complexes constituting the electron transport chain, therefore, the involvement of copper and iron in energy metabolism via their involvement in mitochondrial function (brown adipose tissue activation) is not surprising [29].We performed metal analysis in differentiated adipocytes after treating cells with 1, 50 and 200 µM of menthol during differentiation. We selected few important one based on their importance to adipose tissue health (calcium and cobalt), adipose tissue inflammation (zinc, calcium and iron), mitochondrial activation (iron and copper), cell toxicity (cobalt), glucose utilization and transport (zinc and magnesium), initiation of “*brite*” phenotype in white adipose tissue (iron and copper) for analysis [28,30,31,32,33,34,35]. Menthol administration during differentiation significantly increased the levels of iron and copper at 1 µM whereas there was no significant change in the levels of magnesium, calcium and zinc. Cobalt concentration was significantly decreased in menthol treated differentiated adipocytes. However, at higher doses, 50 and 200 µM (data not shown) there was significant increase in the levels of iron, copper, zinc and magnesium whereas significant decrease in cobalt concentration with no change in calcium concentration. Looking into the metal concentration profile after menthol administration during differentiation of pre-adipocyte to adipocytes, we could suggest that menthol caused mitochondrial activation and increase in “*brite*” phenotype. This data further supports our previous finding where we linked HFD, metal concentration in adipose tissue and menthol administration in an in-vivo model of obesity [14].

**Figure 6:**
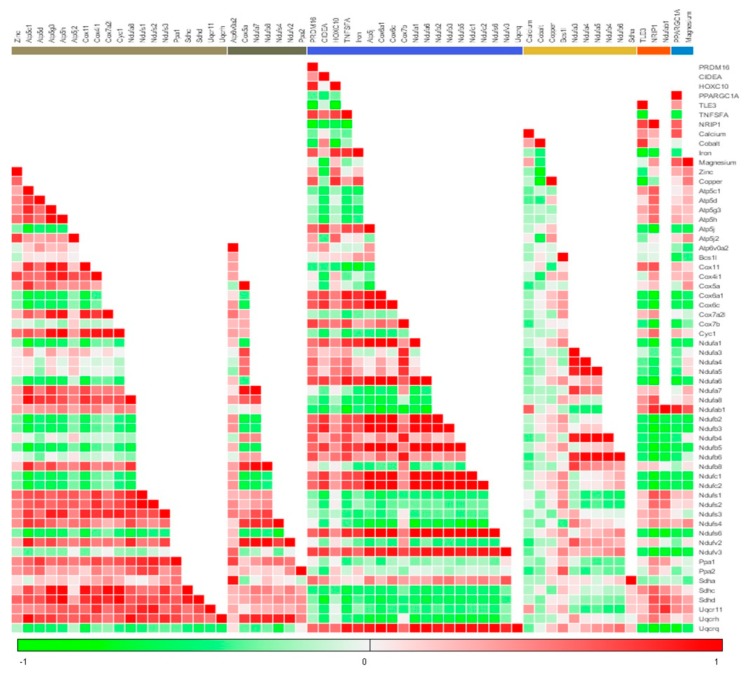
Correlation matrix and clustering of all the data generated through the experiments. The correlation matrix using K-means and Manhattan distance algorithms was drawn, which showed the clustered genes/parameters with respect to each other. Intensity of colours green and red indicate the negative or positive correlation respectively. Genes/parameters along X-axis are clustered using Manhattan distance plot, colour bars showing nearest related groups of genes/parameters.

Based on metal concentration changes, we selected 1 and 50 µM (both 50 and 200 µM had similar metal concentration profile) for further gene expression studies. We studied the change in expression of energy expenditure and “*browning*” related genes in differentiating adipose tissue. By critically looking into the gene expression data we could understand that (a) at 1 µM, menthol administration significantly modulated these genes which is very well corroborated with the existing literature [16], (b) these changes are not dose dependent, as at higher doses 10, 30 and 50 µM of the effect was reversed, although not dose dependently in some of the genes. Although we have not done these experiments but we may speculate that it may be due to desensitization of TRPM8 at this dose due to chronic presence of menthol during the process of adipogenesis. Further, we suggest that menthol at higher concentration might be acting through either TRPM8 dependent or independent mechanisms. There are numerous other actions of menthol which has link with energy expenditure phenomenon like its action of other excitatory or inhibitory channels, its role in TRPM8 independent increase in intracellular calcium, its role in oxidative effects [36]. Importantly at higher doses menthol acts on TRPV3 [37] and TRPA1 channels [38] which are closely related to adipogenesis, glucose utilization, hormone release and “*browning*.” This should be further explored to understand numerous actions of menthol. Based on these effects, we studied the effect of menthol on the mitochondrial activity complexes genes using PCR arrays (84 gene array). The effect on mitochondrial activation genes (approx. 40/84 genes) was positively correlated with energy expenditure genes, significantly higher at 1 µM and at 50 µM, it was decreased, which can be attributed to possible desensitization. In our previous study with TRPV1 agonist, capsaicin, we did see the same phenomenon increase in “*browning*” at 1 µM and decrease at 50 µM, which was correlated to the combination of desensitization and increase in the levels of PPAR-γ at higher doses [23]. We cannot rule out this kind of mechanism with menthol given that both these channels are calcium permeable and in homogenous matrix their agonists can show similar effects. At 1 µM, the changes in metal concentration, especially iron and copper, energy expenditure genes and mitochondrial complex genes are positively correlated (Figure 6). We may say that at 1 µM menthol induces energy expenditure and mitochondrial activation, hence “*brite*” phenotype in differentiating adipocytes. Linking our bioavailable menthol with “*brite*” phenotype induction in differentiating adipocytes, we can suggest that even the topical dose less than 10%, may be 2% or 5% will be sufficient to induce this effect. 

There are some limitations of this study. We could have included further experiments to have a clear picture of desensitization or any other mechanisms responsible for the decrease in effect at 50 µM. We are planning extensive studies along these lines to establish the role of TRPM8 (using pharmacological antagonism), other TRP channels (TRPV3 or TRPA1) or non-TRP channels (calcium channels, GABA/glycine receptors) in menthol’s action. Further about the toxicity of menthol, there are number of human studies both as oral administration as well as topical application (patch) [39,40,41]. Orally LD50 of menthol for rodents was observed approx. 3000 mg/kg of body weight and in canine it is approx. 1 g/kg in cats. The dose which we have used is quite less as compared to these [42]. However, further characterization will enable us to finalize the dose both topically and orally. Also, topical administration at multiple doses should be done to clearly establish cause and effect relationship. These studies will help us to develop topical menthol application as a therapeutic/preventive strategy against obesity and related co-morbidities. 

## 5. Conclusions

**Figure 7:**
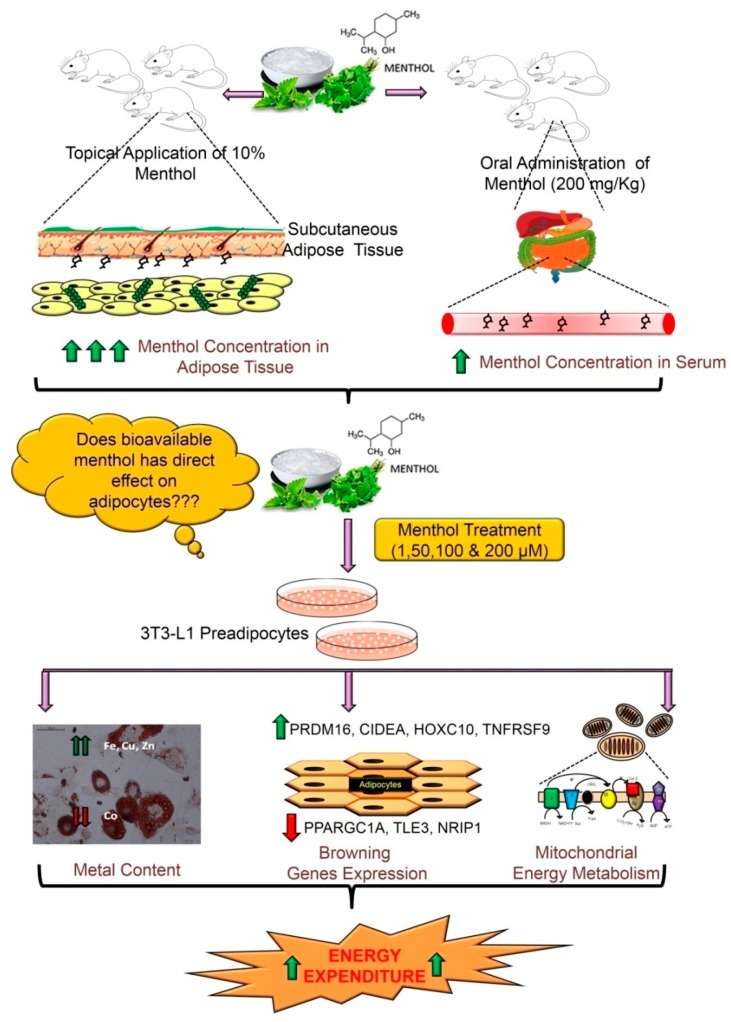
Summary of effect of bioavailable menthol on adipose tissue using in-vitro model. Using the bioavailable concentrations of menthol in adipose tissue and serum upon oral/topical administration, the dosage were decided for in-vitro menthol treatment and effect was checked on expression of genes involved in adipogenesis, *browning*, mitochondrial biogenesis and energy expenditure. The results showed enhanced energy expenditure markers, which indicates improved negative energy balance leading to reduction in obese phenotype.

Over all, summarizing this study, we have provided evidence that a single dose of menthol is sufficiently bioavailable to induce energy expanding “*brite*” phenotype in differentiating adipocytes which is dependent on TRPM8 at a lower concentration (Figure 7). These findings can be further exploited to devise dietary (mint based nutraceuticals) or non-dietary (cold mimicking) approaches to combat obesity and type-2 diabetes. 

## References

[1] WHO Obesity: Preventing and Managing the Global Epidemic. https://www.who.int/nutrition/publications/obesity/WHO_TRS_894/en/.

[2] Mitchell N.S., Catenacci V.A., Wyatt H.R., Hill J.O. (2011). Obesity: Overview of an epidemic. Psychiatr. Clin..

[3] Christiansen E., Garby L. (2002). Prediction of body weight changes caused by changes in energy balance. Eur. J. Clin. Investig..

[4] Donahoo W.T., Levine J.A., Melanson E.L. (2004). Variability in energy expenditure and its components. Curr. Opin. Clin. Nutr. Metab. Care.

[5] Michlig S., Merlini J.M., Beaumont M., Ledda M., Tavenard A., Mukherjee R., Camacho S., Le Coutre J. (2016). Effects of TRP channel agonist ingestion on metabolism and autonomic nervous system in a randomized clinical trial of healthy subjects. Sci. Rep..

[6] Saito M., Okamatsu-Ogura Y., Matsushita M., Watanabe K., Yoneshiro T., Nio-Kobayashi J., Iwanaga T., Miyagawa M., Kameya T., Nakada K. (2009). High incidence of metabolically active brown adipose tissue in healthy adult humans: Effects of cold exposure and adiposity. Diabetes.

[7] van der Lans A.A., Hoeks J., Brans B., Vijgen G.H., Visser M.G., Vosselman M.J., Hansen J., Jörgensen J.A., Wu J., Mottaghy F.M. (2013). Cold acclimation recruits human brown fat and increases nonshivering thermogenesis. J. Clin. Investig..

[8] van Marken Lichtenbelt W., Kingma B., Van Der Lans A., Schellen L. (2014). Cold exposure–an approach to increasing energy expenditure in humans. Trends Endocrinol. Metab..

[9] Romu T., Vavruch C., Dahlqvist-Leinhard O., Tallberg J., Dahlström N., Persson A., Heglind M., Lidell M.E., Enerbäck S., Borga M. (2016). A randomized trial of cold-exposure on energy expenditure and supraclavicular brown adipose tissue volume in humans. Metabolism.

[10] Li C., Li J., Xiong X., Liu Y., Lv Y., Qin S., Liu D., Wei R., Ruan X., Zhang J. (2018). TRPM8 activation improves energy expenditure in skeletal muscle and exercise endurance in mice. Gene.

[11] McKemy D.D., Neuhausser W.M., Julius D. (2002). Identification of a cold receptor reveals a general role for TRP channels in thermosensation. Nature.

[12] Peier A.M., Moqrich A., Hergarden A.C., Reeve A.J., Andersson D.A., Story G.M., Earley T.J., Dragoni I., McIntyre P., Bevan S. (2002). A TRP channel that senses cold stimuli and menthol. Cell.

[13] Bautista D.M., Siemens J., Glazer J.M., Tsuruda P.R., Basbaum A.I., Stucky C.L., Jordt S.-E., Julius D. (2007). The menthol receptor TRPM8 is the principal detector of environmental cold. Nature.

[14] Khare P., Mangal P., Baboota R.K., Jagtap S., Kumar V., Singh D.P., Boparai R.K., Sharma S.S., Khardori R., Bhadada S.K. (2018). Involvement of glucagon in preventive effect of menthol against high fat diet induced obesity in mice. Front. Pharmacol..

[15] Bishnoi M., Kondepudi K.K., Gupta A., Karmase A., Boparai R.K. (2013). Expression of multiple Transient Receptor Potential channel genes in murine 3T3-L1 cell lines and adipose tissue. Pharmacol. Rep..

[16] Jiang C., Zhai M., Yan D., Li D., Li C., Zhang Y., Xiao L., Xiong D., Deng Q., Sun W. (2017). Dietary menthol-induced TRPM8 activation enhances WAT “browning” and ameliorates diet-induced obesity. Oncotarget.

[17] Ma S., Yu H., Zhao Z., Luo Z., Chen J., Ni Y., Jin R., Ma L., Wang P., Zhu Z. (2012). Activation of the cold-sensing TRPM8 channel triggers UCP1-dependent thermogenesis and prevents obesity. J. Mol. Cell Biol..

[18] Rossato M., Granzotto M., Macchi V., Porzionato A., Petrelli L., Calcagno A., Vencato J., De Stefani D., Silvestrin V., Rizzuto R. (2014). Human white adipocytes express the cold receptor TRPM8 which activation induces UCP1 expression, mitochondrial activation and heat production. Mol. Cell. Endocrinol..

[19] Vögler O., Lopez-Bellan A., Alemany R., Tofé S., González M., Quevedo J., Pereg V., Barcelo F., Escriba P. (2008). Structure–effect relation of C18 long-chain fatty acids in the reduction of body weight in rats. Int. J. Obes..

[20] Goralczyk A., van Vijven M., Koch M., Badowski C., Yassin M.S., Toh S.-A., Shabbir A., Franco-Obregón A., Raghunath M. (2017). TRP channels in brown and white adipogenesis from human progenitors: New therapeutic targets and the caveats associated with the common antibiotic, streptomycin. FASEB J..

[21] Khare P., Jagtap S., Jain Y., Baboota R.K., Mangal P., Boparai R.K., Bhutani K.K., Sharma S.S., Premkumar L.S., Kondepudi K.K. (2016). Cinnamaldehyde supplementation prevents fasting-induced hyperphagia, lipid accumulation, and inflammation in high-fat diet-fed mice. Biofactors.

[22] Yang M.T., Fu J., Wang Y.-K., Desai R.A., Chen C.S. (2011). Assaying stem cell mechanobiology on microfabricated elastomeric substrates with geometrically modulated rigidity. Nat. Protoc..

[23] Baboota R.K., Singh D.P., Sarma S.M., Kaur J., Sandhir R., Boparai R.K., Kondepudi K.K., Bishnoi M. (2014). Capsaicin induces “brite” phenotype in differentiating 3T3-L1 preadipocytes. PLoS ONE.

[24] Livak K.J., Schmittgen T.D. (2001). Analysis of relative gene expression data using real-time quantitative PCR and the 2^−ΔΔ*C*T^ method. Methods.

[25] Clemmensen C., Jall S., Kleinert M., Quarta C., Gruber T., Reber J., Sachs S., Fischer K., Feuchtinger A., Karlas A. (2018). Coordinated targeting of cold and nicotinic receptors synergistically improves obesity and type 2 diabetes. Nat. Commun..

[26] Moraes M.N., de Assis L.V.M., dos Santos Henriques F., Batista M.L., Güler A.D., de Lauro Castrucci A.M. (2017). Cold-sensing TRPM8 channel participates in circadian control of the brown adipose tissue. Biochim. Biophys. Acta (BBA) Mol. Cell Res..

[27] Valente A., Carrillo A.E., Tzatzarakis M.N., Vakonaki E., Tsatsakis A.M., Kenny G.P., Koutedakis Y., Jamurtas A.Z., Flouris A.D. (2015). The absorption and metabolism of a single L-menthol oral versus skin administration: Effects on thermogenesis and metabolic rate. Food Chem. Toxicol..

[28] Zhao L., Zhang X., Shen Y., Fang X., Wang Y., Wang F. (2015). Obesity and iron deficiency: A quantitative meta-analysis. Obes. Rev..

[29] Wang C., Liang X., Tao C., Yao X., Wang Y., Wang Y., Li K. (2017). Induction of copper and iron in acute cold-stimulated brown adipose tissues. Biochem. Biophys. Res. Commun..

[30] Severson D.L., Denton R.M., Pask H.T., Randle P.J. (1974). Calcium and magnesium ions as effectors of adipose-tissue pyruvate dehydrogenase phosphate phosphatase. Biochem. J..

[31] Zemel M.B., Shi H., Greer B., Dirienzo D., Zemel P.C. (2000). Regulation of adiposity by dietary calcium. FASEB J..

[32] Yanoff L., Menzie C., Denkinger B., Sebring N., McHugh T., Remaley A., Yanovski J. (2007). Inflammation and iron deficiency in the hypoferremia of obesity. Int. J. Obes..

[33] Prasad A.S., Beck F.W., Bao B., Fitzgerald J.T., Snell D.C., Steinberg J.D., Cardozo L.J. (2007). Zinc supplementation decreases incidence of infections in the elderly: Effect of zinc on generation of cytokines and oxidative stress. Am. J. Clin. Nutr..

[34] Kazi T.G., Afridi H.I., Kazi N., Jamali M.K., Arain M.B., Jalbani N., Kandhro G.A. (2008). Copper, chromium, manganese, iron, nickel, and zinc levels in biological samples of diabetes mellitus patients. Biol. Trace Elem. Res..

[35] Kawakami T., Hanao N., Nishiyama K., Kadota Y., Inoue M., Sato M., Suzuki S. (2012). Differential effects of cobalt and mercury on lipid metabolism in the white adipose tissue of high-fat diet-induced obesity mice. Toxicol. Appl. Pharmacol..

[36] Oz M., El Nebrisi E.G., Yang K.-H.S., Howarth F.C., Al Kury L.T. (2017). Cellular and Molecular Targets of Menthol Actions. Front. Pharmacol..

[37] Macpherson L.J., Hwang S.W., Miyamoto T., Dubin A.E., Patapoutian A., Story G.M. (2006). More than cool: Promiscuous relationships of menthol and other sensory compounds. Mol. Cell. Neurosci..

[38] Karashima Y., Damann N., Prenen J., Talavera K., Segal A., Voets T., Nilius B. (2007). Bimodal action of menthol on the transient receptor potential channel TRPA1. J. Neurosci..

[39] Gelal A., Jacob P., Yu L., Benowitz N.L. (1999). Disposition Kinetics and Effects of Menthol*. Clin. Pharmacol. Ther..

[40] Kaffenberger R.M., Doyle M.J. (1990). Determination of Menthol and Menthol Glucuronide in Human Urine by Gas Chromatography Using an Enzyme-Sensitive Internal Standard and Flame Ionization Detection. J. Chromatogr. B. Biomed. Sci. App..

[41] Martin D., Valdez J., Boren J., Mayersohn M. (2004). Dermal Absorption of Camphor, Menthol, and Methyl Salicylate in Humans. J. Clin. Pharmacol..

[42] JECFA (1999). Menthol. Evaluation of certain food additives and contaminants: forty-second report of the Joint FAO/WHO Expert Committee on Food Additives.

